# Interleukin-17 in disease pathogenesis and therapy: recent advances in targeted drug design and medicinal chemistry

**DOI:** 10.3389/fphar.2026.1836187

**Published:** 2026-07-15

**Authors:** Jing Wen, Fan Liu, Ling Zhang, Linxin Lu, Shaojie Liang, Shufang Du

**Affiliations:** 1 Department of Cariology and Endodontics, State Key Laboratory of Oral Diseases and National Clinical Research Center for Oral Diseases, West China Hospital of Stomatology, Sichuan University, Chengdu, Sichuan, China; 2 Department of Nursing, State Key Laboratory of Oral Diseases and National Clinical Research Center for Oral Diseases, West China Hospital of Stomatology, Sichuan University, Chengdu, Sichuan, China; 3 State Key Laboratory of Oral Diseases and National Center for Stomatology and National Clinical Research Center for Oral Diseases and Department of West Outpatient, West China Hospital of Stomatology, Sichuan University, Chengdu, Sichuan, China; 4 Department of Orthodontics, State Key Laboratory of Oral Diseases and National Clinical Research Center for Oral Diseases, West China Hospital of Stomatology, Sichuan University, Chengdu, Sichuan, China

**Keywords:** inflammatory diseases, inhibitors, interleukin-17, monoclonal antibodies, small molecule inhibitors

## Abstract

Interleukin-17 (IL-17) is a key pro-inflammatory cytokine that plays an essential role in host defense and immune regulation. Dysregulated IL-17 expression and signaling are implicated in the pathogenesis of a broad range of chronic inflammatory and autoimmune diseases, including psoriasis, systemic lupus erythematosus, periodontitis, and cancer. Increasing insights into IL-17 receptor engagement and downstream signaling networks, have established the IL-17 axis as a promising therapeutic target. Monoclonal antibodies targeting IL-17 or its receptor have demonstrated substantial clinical efficacy; however, their widespread application is limited by high cost, suboptimal tissue penetration, potential immunogenicity, and therapy-associated adverse effects. These limitations have driven growing interest in the development of alternative therapeutic strategies. This review provides a comprehensive overview of IL-17 signaling pathways and their contributions to disease pathogenesis and systematically summarizes current therapeutic approaches targeting the IL-17 axis, including monoclonal antibodies and emerging small-molecule inhibitors. Collectively, these insights offer a conceptual framework to support the rational design and development of next-generation IL-17-targeted therapeutics.

## 1 Introduction

The interleukin-17 (IL-17) family consists of six structurally homologous isoforms, designated IL-17A through IL-17F, which are indispensable for maintaining mucosal barrier integrity and immune homeostasis (Huangfu et al., 2023). Aberrant expression of IL-17 cytokines is profoundly implicated in various pathophysiological cascades, including chronic inflammation, autoimmune disorders, and oncogenesis. Given its central role in orchestrating immune-mediated tissue damage, the IL-17 signaling axis has emerged as a pivotal driver of disease progression and a high-value therapeutic target for pharmacological intervention (Huangfu et al., 2023; Mills, 2023; Sánchez-Rodríguez and Puig, 2023; Vitiello and Miller, 2020).

Currently, four anti-IL-17 monoclonal antibodies—secukinumab, ixekizumab, brodalumab, and bimekizumab—have received regulatory approval in major international markets, including the United States, Europe, Australia, and several Asian countries (Table 1) (AbuHilal et al., 2016; Liu L. et al., 2016; Singh et al., 2020). In contrast, other IL-17-targeting antibodies, such as netakimab, vunakizumab, and xeligekimab, are approved only in specific countries or regions (e.g., Russia or China) and have not received approval from the FDA or EMA. Consequently, the global clinical experience and regulatory status of these agents differ substantially. These biologics are administered via intravenous, subcutaneous, or intramuscular routes of injection rather than oral administration. Owing to their prolonged half-lives, monoclonal antibodies (mAbs) require infrequent dosing, which enhances treatment convenience and may improve patient adherence (Posner et al., 2019). However, their large molecular size limits their ability to cross the blood–brain barrier, thereby restricting their utility in central nervous system disorders (Gautam et al., 2023; Wu et al., 2023). Additionally, another disadvantage of mAbs therapies is the high cost associated with their production, storage, and administration (Alejandra et al., 2023).

**TABLE 1:** Globally approved anti-IL-17 biologics.

Name	FDA-approved for use date	Target	Indications at the time of FDA approval	Indications	Application number
Secukinumab	01/21/2015	IL-17A	Treatment of moderate to severe plaque psoriasis in adult patients who are candidates for systemic therapy or phototherapy and who did not respond well to medication applied directly to the skin.	Psoriasis Rheumatoid arthritis Ankylosing spondylitis Psoriatic arthritis Asthma Multiple sclerosis Type 1 Diabetes Crohn’s disease	125504
Ixekizumab	03/22/2016	IL-17A	Treatment of moderate to severe plaque psoriasis in adult patients who are candidates for systemic therapy or phototherapy	Psoriasis Rheumatoid arthritis	125521
Brodalumab	02/15/2017	IL-17RA	Treatment of moderate to severe plaque psoriasis in adults who may benefit from systemic treatment (such as injections or pills) or phototherapy (ultraviolet light treatment) and who did not respond or lost response to other systemic treatments	Psoriasis Psoriatic arthritis Asthma Crohn’s disease	761032
Bimekizumab	17/10/2023	IL-17A/IL-17F	Treatment of moderate to severe plaque psoriasis in adult patients who are candidates for systemic therapy or phototherapy.	Psoriasis Ankylosing spondylitis Non-radiographic axial spondyloarthritis Psoriatic arthritis Hidradenitis suppurativa	761151

In contrast, small-molecule therapeutics offer distinct pharmacological advantages, including oral bioavailability, improved tissue distribution, and greater flexibility in dose optimization. These properties make them attractive alternatives or complement to antibody-based therapies. However, the development of small-molecule inhibitors targeting IL-17 signaling remains challenging. As a cytokine-driven pathway, IL-17 signaling is largely mediated by protein–protein interactions (PPI), which typically involve large and relatively flat binding interfaces that are difficult to modulate using conventional small molecules (Allen and Lumb, 2020). Moreover, given the critical role of IL-17 in host defense, therapeutic inhibition of this pathway raises concerns regarding increased susceptibility to infections and potential disruption of immune homeostasis.

Despite these challenges, recent advances in medicinal chemistry have facilitated the identification of novel strategies for targeting the IL-17 signaling pathway. While several recent reviews have summarized the biological functions of IL-17 and the clinical progress of antibody-based therapies, systematic integration of IL-17 signaling mechanisms with advances in targeted drug design and medicinal chemistry—particularly in the context of small-molecule inhibitors—remains limited (Kim et al., 2025; Koh et al., 2024). In this review, we provide a comprehensive overview of IL-17 signaling pathways and their roles in disease pathogenesis, followed by a detailed discussion of current therapeutic strategies targeting the IL-17 axis, including monoclonal antibodies, small-molecule inhibitors, and emerging non-traditional modalities. By bridging immunological mechanisms with pharmacological innovation, this review aims to inform the rational design and development of next-generation IL-17-targeted therapeutics.

## 2 IL-17 signaling pathways

**FIGURE 1:**
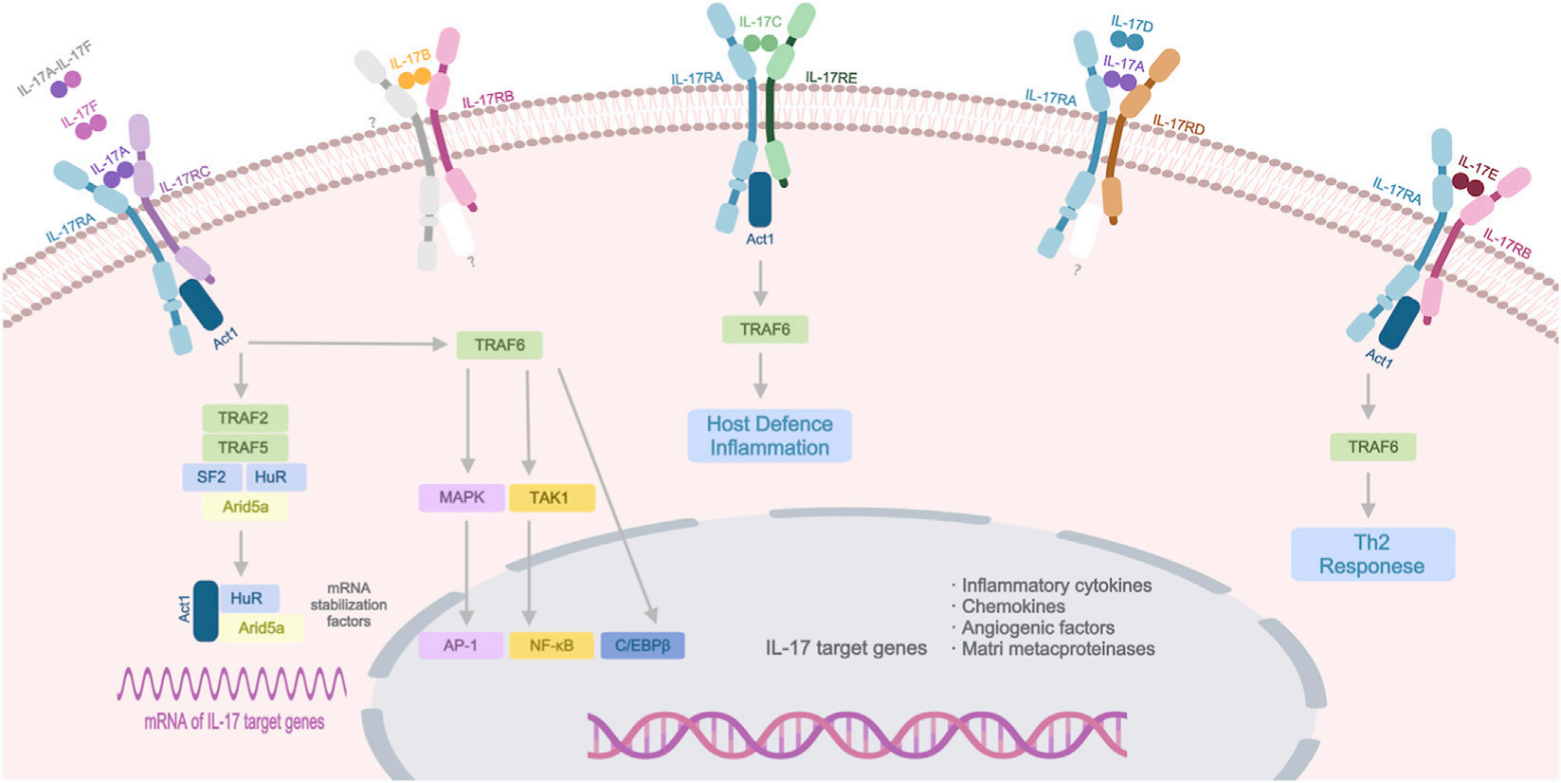
IL-17 receptor signaling network. The IL-17R family (IL-17RA–IL-17RE) utilizes intracellular SEFIR domains to recruit Act1 upon ligand binding. Act1 engages TRAF6 to activate the TAK1 complex, driving downstream NF-κB, MAPK, and C/EBP transcriptional pathways to induce pro-inflammatory gene expression. Post-transcriptionally, Act1 coordinates with RNA-binding proteins to regulate mRNA stability or decay, while feedback loops (via CBAD, A20, and TRAF3/4) tightly control pathway magnitude. The schematic diagram in this figure was created using MedPeer.

The interleukin-17 receptor (IL-17R) family comprises five subunits: IL-17RA, IL-17RB, IL-17RC, IL-17RD, and IL-17RE, with IL-17RA first identified in 1995. Each receptor contains fibronectin type I and II motifs and a SEFIR domain, which are essential for intracellular signaling (Figure 1). IL-17RA also possesses a C/EBPβ activation domain (CBAD), which modulates downstream signaling (Rex et al., 2023). Distinct receptor subunits form heterodimers on the cell surface to enable ligand recognition. For example, IL-17A and IL-17F signal predominantly through IL-17RA/IL-17RC complexes, while IL-17C binds to IL-17RA/IL-17RE complexes (Singh et al., 2020). IL-17RD acts as a co-receptor for IL-17A but not IL-17F (Liu et al., 2020). Although IL-17B interacts with IL-17RB, its intracellular signaling remains poorly understood.

Ligand binding to IL-17R recruits Act1 via the SEFIR domain, enabling interaction with tumor necrosis factor receptor-associated factors (TRAFs). This process activates nuclear factor κB (NF-κB), mitogen-activated protein kinases (MAPKs), and CCAAT/enhancer-binding proteins (C/EBPs), thereby inducing the expression of pro-inflammatory genes (Wilson et al., 2022). Act1 also functions as an E3 ubiquitin ligase and RNA-binding protein, mediating both transcriptional and post-transcriptional regulation (Liu et al., 2009; Meehan and Wang, 2022). Negative feedback is provided by CBAD-mediated recruitment of TRAF3 and A20 (Huangfu et al., 2023).

The IL-17 pathway shares features with TLR/IL-1R signaling (Goepfert et al., 2022). Act1, a dormant adaptor in TLR/IL-1R signaling pathways, plays a central role in IL-17 signaling (Hong et al., 2024). Upon binding to IL-17R, Act1 activates its E3 ligase activity, leading to ubiquitination of TRAF6, and initiates NF-κB and MAPK cascades (Lipovsky et al., 2021). This ubiquitination recruits the TAK1 complex, which activates the IKK complex, leading to NF-κB phosphorylation and transcription of pro-inflammatory genes (Shibabaw et al., 2023).

In the IL-17–driven NF-κB cascade, IκBζ regulates target gene expression, with its production enhanced by IL-17 through transcriptional and translational control (Yamazaki et al., 2022). IL-17 also suppresses miR-23b to facilitate gene induction. In the MAPK pathway, IL-17 activates p38 and JNK via the TPL2–TAK1 axis, which further promotes IKK activation and downstream gene expression (Rex et al., 2023).

In IL-17 signaling, IκBζ and C/EBP transcription factors share redundant promoter-binding sites, enhancing inflammatory responses (Karlsen et al., 2010; Muromoto et al., 2022). Because excessive activation can cause tissue damage, TRAF6-mediated cascades are tightly regulated (Liu Y. et al., 2023). A20, induced by NF-κB, deubiquitinates TRAF6, thereby inhibiting downstream signaling, while USP25, together with TRAF3 and TRAF4, suppresses IL-17-driven inflammation by competing with TRAF6 (Aslani et al., 2022).

Emerging evidence suggests that IL-17-mediated induction of IκBζ may contribute to metabolic reprogramming in fibroblast reticular cells, potentially through regulation of CPT1A expression (Majumder et al., 2019; Mueller, 2019; Wang and Ding, 2022). Certain metabolic intermediates have been reported to suppress IL-17-induced IκBζ activation, further highlighting the close interplay between cellular metabolism and immune regulation (Yamazaki et al., 2022).

The IL-17 signaling cascade not only activates the NF-κB and MAPK pathways to induce inflammatory gene transcription but also regulates post-transcriptional mRNA stability. IL-17-induced transcripts generated via NF-κB and MAPK pathways are inherently unstable due to interactions with RNA-binding proteins (RBPs) at the 3′ untranslated region (3′UTR) (Behrens and Heissmeyer, 2022). Stabilizers such as HuR, Act1, ARID-5A, and DDX3X enhance persistence, whereas SF-2 and Regnase-1 accelerate decay, thereby modulating the magnitude of IL-17-driven inflammatory responses (Ohgakiuchi et al., 2020; Vidal et al., 2021).

IL-17 modulates inflammation both directly via its receptor and indirectly through crosstalk with other cytokines and non-cytokine mediators, including EGFR, FGF2, CARD14, and NOTCH, thereby affecting tissue repair, autoimmunity, and tumorigenesis (Corrado et al., 2022; Lee et al., 2023). In colonic epithelium and psoriasis, IL-17 synergizes with FGF2 or CARD14/TRAF6 to enhance ERK1/2 or NF-κB signaling, highlighting therapeutic potential of IL-17-targeting antibodies (Miossec, 2021).

## 3 IL-17 associated conditions

**FIGURE 2:**
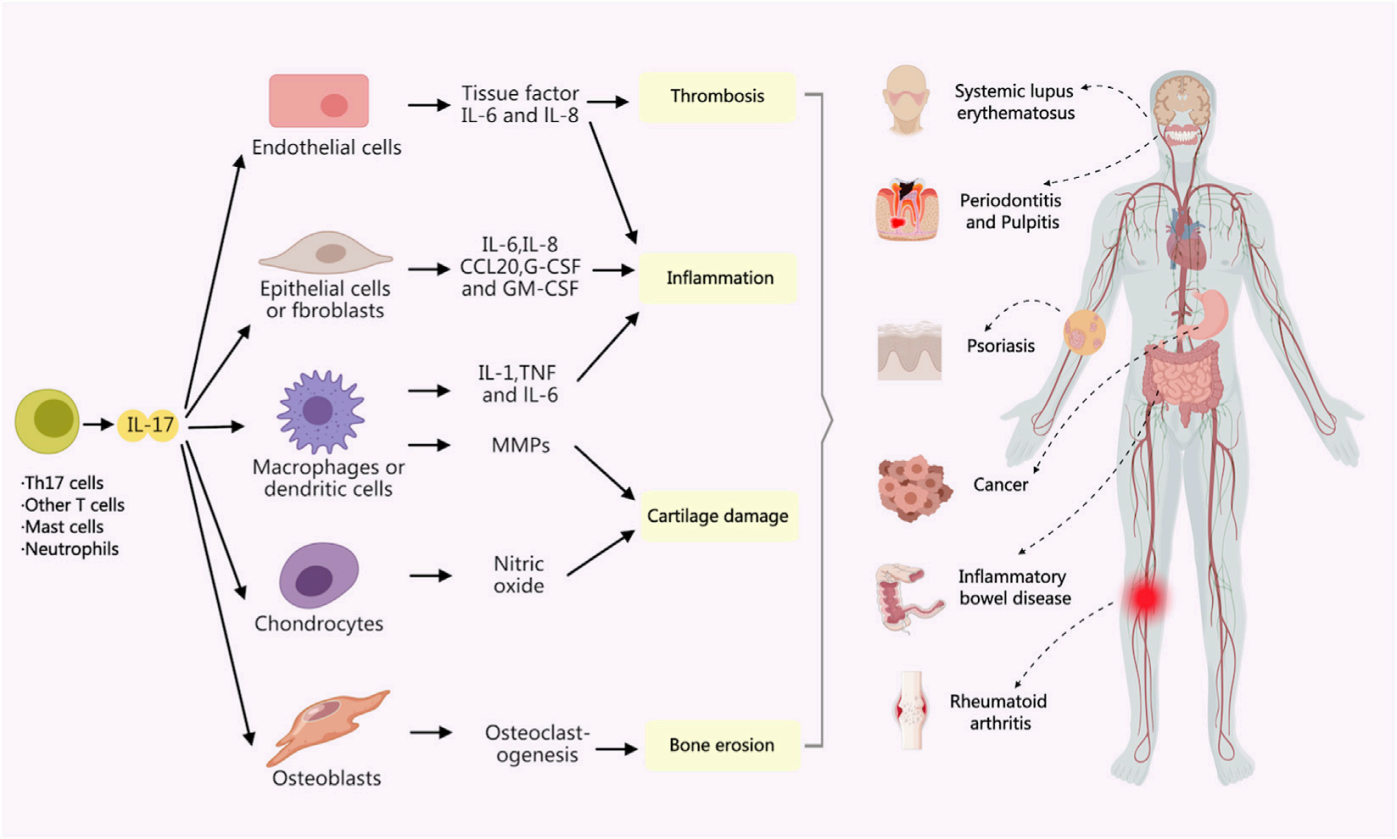
Pathophysiological roles of IL-17 in target cell activation and systemic diseases. IL-17, secreted by Th17, mast, and epithelial cells, acts on diverse target cells (endothelial, epithelial, macrophages, chondrocytes, and osteoblasts) to induce effector molecules including cytokines (IL-6, IL-8, TNF, IL-1), chemokines, MMPs, and nitric oxide. These downstream factors mediate clinical manifestations such as thrombosis, inflammation, cartilage damage, and bone erosion, culminating in various systemic disorders including periodontitis, pulpitis, psoriasis, cancer, inflammatory bowel disease, and rheumatoid arthritis. The schematic diagram in this figure was created using MedPeer.

Increased IL-17 expression has been strongly linked to the development and progression of numerous pathological conditions, such as psoriasis, systemic lupus erythematosus, inflammatory bowel disease, periodontitis, pulpitis, cancer, and others (Figure 2).

### 3.1 Psoriasis

Psoriasis is a chronic systemic inflammatory disease influenced by genetic, environmental, metabolic, and immune factors (Liu et al., 2024). IL-17 is central to its pathogenesis, with IL-17 receptor–associated molecules such as CMTM4 contributing to keratinocyte-driven inflammation (Knizkova et al., 2022). Th17 cells recognize lipid antigens via CD1a, further amplifying cutaneous immune activation (Ye et al., 2024). IL-17E (IL-25) is elevated in lesions and promotes keratinocyte proliferation, while its deletion attenuates disease severity in murine models (Gangwar et al., 2022). Crucially, the inflammatory cascade triggered by IL-17 extends beyond the cutaneous boundary to drive psoriatic arthritis (PsA), a heterogeneous systemic condition characterized by peripheral arthritis, enthesitis, dactylitis, and axial involvement. Within the inflamed synovium and entheseal microenvironments of PsA patients, markedly elevated expressions of both IL-17A and IL-17F stimulate synovial fibroblasts to release downstream pro-inflammatory cytokines and matrix metalloproteinases, accelerating tissue degradation and joint damage (Sánchez-Rodríguez and Puig, 2023). IL-17–targeting biologics, including secukinumab, brodalumab, bimekizumab, and netakimab, provide superior clinical efficacy across both dermatological and musculoskeletal domains but may increase infection risk (Oliver et al., 2022; Puig et al., 2021).

### 3.2 Systemic lupus erythematosus

Systemic lupus erythematosus (SLE) is a systemic autoimmune disease marked by multi-organ injury, autoantibody production, and immune complex deposition (Yang et al., 2023). IL-17, mainly secreted by Th17 cells, contributes to disease progression by cooperating with IL-21, IL-22, and TNF-α to amplify inflammation (Kini et al., 2022). Disruption of the Th17/Treg balance and IL-23–driven Th17 differentiation further enhances immune activation (Huang et al., 2024). IL-17 also promotes plasma cell survival through p38 signaling, sustaining autoimmunity (Ma et al., 2021; Pöysti et al., 2021). Although IL-23 or IL-12/23 inhibitors show partial efficacy, IL-17 levels are not consistently reduced (Rafael-Vidal et al., 2020; Robert and Miossec, 2020). Emerging strategies targeting Th17 differentiation or indirectly modulating IL-17, including agents such as voclosporin and anifrolumab, offer therapeutic potential (Kalunian et al., 2023; Rovin et al., 2021).

### 3.3 Hidradenitis suppurativa

Hidradenitis suppurativa (HS) is a chronic, relapsing inflammatory skin disorder characterized by painful nodules, abscesses, and sinus tract formation in intertriginous regions (Sabat et al., 2025). Increasing evidence implicates the IL-17/Th17 axis as a central driver of its pathogenesis. Lesional HS skin shows marked upregulation of IL-17A, IL-17F, and related cytokines, accompanied by infiltration of Th17 cells, neutrophils, and innate immune cells. IL-17 promotes keratinocyte activation, enhances production of pro-inflammatory mediators such as TNF-α, IL-1β, and chemokines, and sustains neutrophil recruitment, thereby amplifying chronic inflammation and tissue destruction. Dysbiosis of the skin microbiome may further augment IL-17–mediated immune responses. Clinically, the therapeutic relevance of this pathway is supported by the efficacy of IL-17 inhibitors (Gao et al., 2024).

### 3.4 Inflammatory bowel disease

Inflammatory bowel disease (IBD), including ulcerative colitis and Crohn’s disease, is a chronic relapsing condition driven by dysregulated intestinal immunity (Kumar et al., 2024). IL-17, produced by Th17 cells and ILCs, is elevated in inflamed mucosa and acts synergistically with IL-23 and IFN-γ to promote inflammation. Genetic studies link IL-17B/IL-17E/IL-17RB to ulcerative colitis and IL-17C/IL-17RC to Crohn’s disease (Cai et al., 2023). IL-17 and IL-22 also support epithelial barrier integrity, underscoring their dual protective and pathogenic functions (Akuzum and Lee, 2022). Clinically, IL-17 blockade may exacerbate inflammatory bowel diseases, particularly Crohn’s disease and ulcerative colitis, likely owing to the protective role of IL-17 in maintaining intestinal epithelial barrier integrity, whereas IL-12/23-targeted therapies have demonstrated clinical benefit (Caron et al., 2022). Nevertheless, IL-17 inhibitors demonstrate favorable safety profile in patients with psoriasis, and AS, with a low incidence of new-onset or exacerbated IBD events in clinical trials (Tang et al., 2018).

### 3.5 Periodontitis

Periodontitis (PD) is a chronic inflammatory disease marked by progressive destruction of tooth-supporting tissues, initiated by dental plaque and aggravated by dysregulated immune responses (Tanwar et al., 2024). IL-17A and Th17 cells are significantly upregulated in PD, accompanied by reduced Treg cells, reflecting an imbalanced local immune microenvironment (Alarcón-Sánchez et al., 2024; McClure et al., 2024). IL-17A drives inflammation by stimulating fibroblasts, macrophages, and epithelial cells to release pro-inflammatory mediators and promotes bone resorption through RANKL-mediated osteoclastogenesis (Tan et al., 2021; Techatanawat et al., 2020). Detected in gingival tissues and fluids, IL-17A also enhances neutrophil recruitment and adaptive immune activation, underscoring its central pathogenic role (Irie et al., 2023; Mazurek-Mochol et al., 2023). Beyond conventional mechanical debridement, targeting the IL-17 axis presents a conceptual paradigm for host modulation in severe PD. However, direct clinical evidence evaluating anti-IL-17 biologics specifically for periodontitis remains absent. Complete systemic inhibition of IL-17 risks compromising oral mucosal immunity against opportunistic infections (e.g., *Candida* albicans). Thus, future clinical translation will likely require site-specific delivery or localized small-molecule formulation rather than systemic blockade.

### 3.6 Pulpitis

Pulpitis is an inflammatory disease of the dental pulp, commonly caused by bacterial infection, and may progress to apical periodontitis or maxillofacial infections due to the limited collateral circulation within the dental pulp tissue (Donnermeyer et al., 2023). Microbial products activate immune and non-immune cells, inducing the release of cytokines and chemokines, which contribute to the establishment of a complex and dynamic inflammatory network (Wu et al., 2026). IL-17 is a key mediator of this inflammatory process, persistently elevated during pulpitis and promoting the production of IL-6, IL-8, and MMPs, thereby enhancing collagen degradation and activating MAPK signaling pathways that drive tissue destruction (Andujar et al., 1991; Kritikou et al., 2021). It also induces CCL2-mediated monocyte recruitment and their differentiation into macrophages, further amplifying the local inflammatory response. Collectively, these findings highlight IL-17’s pivotal role in pulpitis and its therapeutic potential as a promising target for intervention. Collectively, these findings highlight IL-17’s pivotal role in orchestrating pulpal inflammation. Nevertheless, translating these mechanisms into dental therapies faces strict anatomical hurdles. Due to the low-compliance environment of the enclosed pulp chamber, systemic administration of current macromolecular inhibitors is pharmacologically impractical for local pulpitis. To date, no clinical trials have validated anti-IL-17 therapies in endodontics, necessitating advanced biomaterial-based local targeting before clinical evaluation.

### 3.7 Cancer

IL-17 is critically involved in both the initiation and progression of multiple cancers, where it promotes tumor growth through the regulation of angiogenesis, enhanced cellular proliferation, inhibition of apoptosis, and modulation of anti-tumor immune responses (Song et al., 2023). Elevated IL-17 levels have been reported in breast, gastric, ovarian, and colorectal cancers, and are positively associated with increased microvascular density, primarily through VEGF- and IL-17/IL-17RA–mediated signaling pathways (Popović et al., 2023; Song et al., 2021).

IL-17 also promotes chemoresistance and supports cancer stem cell maintenance through the NF-κB and MAPK signaling pathways, while facilitating metastasis via IL-6–mediated STAT3 activation in lung cancer (Liu L. et al., 2023). However, IL-17 may also exert context-dependent tumor-suppressive effects, as higher expression levels have been associated with improved survival in colorectal cancer, and IL-17D promotes the recruitment of NK cells to mediate anti-tumor immune responses (Yang et al., 2022). Collectively, these findings highlight IL-17’s dual and context-dependent roles in tumor biology, underscoring its potential as a therapeutic target in cancer treatment.

### 3.8 Others

IL-17 is implicated in a broad range of immune-mediated diseases. In rheumatoid arthritis (RA), IL-17A contributes to synovial inflammation by inducing the production of IL-6, IL-8, G-CSF, and CCL20, thereby promoting neutrophil recruitment and joint destruction (Zhao et al., 2023). Although IL-17 has been implicated in RA pathogenesis, clinical trials evaluating IL-17-targeted therapies, including secukinumab, ixekizumab, and the dual TNF-α/IL-17A antibody ABT-122, have generally failed to demonstrate sufficient efficacy compared with established therapeutic options (Fleischmann et al., 2017; Genovese et al., 2018).

In ankylosing spondylitis, activation of the IL-23/IL-17 signaling axis drives enthesitis and structural damage (Fu et al., 2023). Recent clinical paradigms have extended this pathological framework across the entire spectrum of axial spondyloarthritis (axSpA), encompassing both radiographic axial spondyloarthritis (r-axSpA) and non-radiographic axial spondyloarthritis (nr-axSpA). In these subtypes, elevated concentrations of IL-17A and IL-17F within the entheseal microenvironment promote aberrant osteoblast differentiation and subsequent new bone formation (Shah et al., 2020). IL-17 blockade—including secukinumab, ixekizumab, and the dual IL-17A/IL-17F inhibitor bimekizumab—has demonstrated robust therapeutic efficacy, and the small-molecule agent iguratimod may modulate IL-17–mediated inflammation and bone remodeling (Liu et al., 2021; van der Heijde et al., 2018). Accumulating long-term evidence underlines the efficacy of these agents in achieving sustained reductions in magnetic resonance imaging (MRI) sacroiliac joint inflammation and halting radiographic progression. Furthermore, emerging data indicate that dual inhibition of IL-17A and IL-17F confers a significant protective effect against acute anterior uveitis (AAU), a prevalent extra-articular manifestation, markedly lowering recurrence rates in high-risk patients and broadening the therapeutic utility of the IL-17 signalosome in axSpA management (Baraliakos et al., 2024).

Beyond axial spondyloarthritis, emerging clinical evidence has extended the therapeutic paradigm of IL-17 inhibitors to large-vessel vasculitis and related rheumatic conditions. In giant cell arteritis (GCA) and polymyalgia rheumatica (PMR), dysregulated Th17 responses and elevated systemic IL-17A levels are critically involved in vascular wall inflammation and systemic ischemic manifestations (Zeisbrich et al., 2023). Recent clinical trials evaluating secukinumab in GCA and PMR have met their primary endpoints, demonstrating significant efficacy in achieving sustained remission and reducing cumulative glucocorticoid requirements. These developments suggest that the IL-17 signaling cascade represents a highly viable pathophysiological node for treating refractory vascular and systemic rheumatic diseases (Nepal et al., 2023; Sun and Pope, 2025).

In systemic sclerosis, IL-17A produced by Th17 cells contributes to fibroblast activation and collagen deposition, although context-dependent anti-fibrotic effects have also been reported (Wei et al., 2022). However, evidence supporting IL-17-targeted therapy in systemic sclerosis remains preliminary and limited. Increasing evidence also implicates IL-17-driven neuroinflammation in psychiatric disorders, where dysregulated Th17 responses and elevated pro-inflammatory cytokines are frequently observed, suggesting a potential role of IL-17 in immune dysregulation and microbiota–gut–brain axis disturbances (Lu et al., 2023). Importantly, although IL-17 dysregulation has been implicated in a variety of immune-mediated diseases, therapeutic blockade of IL-17 has not demonstrated adequate clinical efficacy in rheumatoid arthritis, asthma, diabetes mellitus, or multiple sclerosis. Furthermore, IL-17 inhibition may aggravate inflammatory bowel diseases, including Crohn’s disease and ulcerative colitis, highlighting the context-dependent biological functions of IL-17 across different tissues and disease settings.

## 4 IL-17-targeted biopharmaceuticals

Over the past 2 decades, extensive research and development targeting the interleukin-17 (IL-17) signaling cascade have revolutionized the therapeutic landscape of chronic inflammatory and autoimmune diseases. To date, only four anti-IL-17 monoclonal antibodies—namely secukinumab, ixekizumab, bimekizumab, and brodalumab—have successfully navigated rigorous clinical evaluation to achieve widespread regulatory approvals in major international jurisdictions, including Europe, the United States, Australia, and several Asian countries. These agents currently constitute the cornerstone of global clinical experience in IL-17-targeted biological therapy. In contrast, several other newly developed monoclonal antibodies and emerging peptide-based inhibitors have either secured conditional or full regulatory approvals restricted to specific countries or regions (e.g., netakimab in Russia; vunakizumab and xeligekimab in China), or remain in active global clinical pipelines. Crucially, these regional biologics have not yet received marketing authorization from major global regulatory agencies such as the U.S. Food and Drug Administration (FDA) or the European Medicines Agency (EMA). Consequently, their global clinical utility, long-term safety profiles, and regulatory classifications differ substantially from the internationally established standard-of-care biologics. In the following sections, we provide a comprehensive and detailed overview of these biopharmaceuticals, systematically characterizing their molecular structures, mechanisms of action, clinical trial profiles, and distinct regulatory statuses (Table 2).

**TABLE 2:** Monoclonal antibody inhibitors of the IL-17 pathway.

Drug	NCT number	Study status	Conditions	Interventions	Sponsor	Phases	Study type	Start date
Vunakizumab	NCT06881290	RECRUITING	Lupus Erythematosus, Systemic	DRUG: Vunakizumab (IL-17A inhibitor)	Chinese SLE treatment And Research Group	PHASE1	INTERVENTIONAL	2025/5/19
	NCT06779097	NOT_YET_RECRUITING	Moderate to Severe Plaque Psoriasis	DRUG: Vunakizumab (IL-17A inhibitor)	Suzhou Suncadia Biopharmaceuticals Co., Ltd.	PHASE4	INTERVENTIONAL	Feb-25
	NCT07069270	NOT_YET_RECRUITING	Vunakizumab|Adebrelimab|Small Cell Lung Cancer	DRUG: Vunakizumab (IL-17A inhibitor)|DRUG: Adebrelimab|DRUG: Chemotherapy	Fujian Cancer Hospital	PHASE2	INTERVENTIONAL	2025/9/1
	NCT06770088	NOT_YET_RECRUITING	Spondyloarthritis (SpA)	DRUG: vnacicizumab	Sun Yat-sen University	PHASE4	INTERVENTIONAL	2025/1/10
Xeligekimab	NCT06802848	NOT_YET_RECRUITING	Plaque Psoriasis|Psoriatic Arthritis|Scalp Psoriasis|Nail Psoriasis|Palmoplantar Psoriasis|Genital Psoriasis	DRUG: Xeligekimab injection	Chongqing Genrix Biopharmaceutical Co., Ltd	​	OBSERVATIONAL	2025/1/30
Bimekizumab	NCT04340076	ACTIVE_NOT_RECRUITING	Psoriasis|Psoriasis Vulgaris	DRUG: Secukinumab|DRUG: Ixekizumab|DRUG: Brodalumab|DRUG: Guselkumab|DRUG: Risankizumab|DRUG: Tildrakizumab|DRUG: Bimekizumab	Radboud University Medical Center	PHASE4	INTERVENTIONAL	2020/8/20
	NCT06336343	RECRUITING	Plaque Psoriasis	DRUG: Bimekizumab	Icahn School of Medicine at Mount Sinai	PHASE4	INTERVENTIONAL	2024/6/3
Netakimab	NCT07008547	ACTIVE_NOT_RECRUITING	Plaque Psoriasis	BIOLOGICAL: Netakimab|DRUG: Placebo	SPH-BIOCAD (HK) Limited	PHASE3	INTERVENTIONAL	2024/7/30
	NCT06640517	ACTIVE_NOT_RECRUITING	Plaque Psoriasis	DRUG: Netakimab|DRUG: Placebo|DRUG: Adalimumab	Biocad	PHASE3	INTERVENTIONAL	2024/8/8
	NCT03447704	COMPLETED	Ankylosing Spondylitis	DRUG: BCD-085|OTHER: placebo	Biocad	PHASE3	INTERVENTIONAL	2018/2/9
Ixekizumab	NCT04340076	ACTIVE_NOT_RECRUITING	Psoriasis|Psoriasis Vulgaris	DRUG: Secukinumab|DRUG: Ixekizumab|DRUG: Brodalumab|DRUG: Guselkumab|DRUG: Risankizumab|DRUG: Tildrakizumab|DRUG: Bimekizumab	Radboud University Medical Center	PHASE4	INTERVENTIONAL	2020/8/20
	NCT06945107	RECRUITING	Plaque Psoriasis	DRUG: Picankibart|DRUG: Secukinumab|DRUG: Placebo|DRUG: Ixekizumab	Innovent Biopharmaceutical Technology (Hangzhou) Co., LTD.	PHASE3	INTERVENTIONAL	2025/5/27
	NCT04724629	COMPLETED	COVID-19	BIOLOGICAL: Ixekizumab|BIOLOGICAL: Aldesleukin|DRUG: Colchicine|DRUG: Standard of care (SOC)	University of Sao Paulo	PHASE3	INTERVENTIONAL	2021/1/5
	NCT04537689	RECRUITING	Plaque Psoriasis	BIOLOGICAL: Ixekizumab|DRUG: Methotrexate|DRUG: Cyclosporin A|DRUG: Acitretin	Singapore General Hospital	PHASE4	INTERVENTIONAL	2020/12/10
	NCT06398106	RECRUITING	Psoriasis Vulgaris	DRUG: Proactive TDM-based dosing of secukinumab|DRUG: Proactive TDM-based dosing of ixekizumab|DRUG: Proactive TDM-based dosing of guselkumab	University Hospital, Ghent	PHASE4	INTERVENTIONAL	2024/12/20
Secukinumab	NCT04967950	UNKNOWN	Psoriatic Arthritis	DRUG: Secukinumab 300 MG|DRUG: Secukinumab 150 MG|DRUG: Methotrexate	Peking Union Medical College Hospital	PHASE1	INTERVENTIONAL	2021/7/15
	NCT04340076	ACTIVE_NOT_RECRUITING	Psoriasis|Psoriasis Vulgaris	DRUG: Secukinumab|DRUG: Ixekizumab|DRUG: Brodalumab|DRUG: Guselkumab|DRUG: Risankizumab|DRUG: Tildrakizumab|DRUG: Bimekizumab	Radboud University Medical Center	PHASE4	INTERVENTIONAL	2020/8/20
	NCT06945107	RECRUITING	Plaque Psoriasis	DRUG: Picankibart|DRUG: Secukinumab|DRUG: Placebo|DRUG: Ixekizumab	Innovent Biopharmaceutical Technology (Hangzhou) Co., LTD.	PHASE3	INTERVENTIONAL	2025/5/27
	NCT05891964	COMPLETED	Plaque Psoriasis	DRUG: Secukinumab Injection	Jinnah Postgraduate Medical Centre	PHASE4	INTERVENTIONAL	2021/7/5
	NCT06833112	NOT_YET_RECRUITING	Ankylosing Spondylitis	DRUG: Secukinumab|DRUG: Adalimumab	The Affiliated Hospital of Guizhou Medical University	PHASE4	INTERVENTIONAL	Feb-25
	NCT03623867	COMPLETED	Psoriatic Arthritis	DRUG: Secukinumab|DRUG: Placebo	Chinese University of Hong Kong	PHASE4	INTERVENTIONAL	2020/5/18
	NCT04237116	TERMINATED	Plaque Psoriasis|Non-alcoholic Fatty Liver Disease	BIOLOGICAL: Investigational Arm - secukinumab|BIOLOGICAL: Control Arm - placebo	Novartis Pharmaceuticals	PHASE3	INTERVENTIONAL	2020/2/19
	NCT04717466	COMPLETED	Psoriasis|Healthy	BIOLOGICAL: Secukinumab	University of Miami	PHASE4	INTERVENTIONAL	2021/6/29
	NCT06398106	RECRUITING	Psoriasis Vulgaris	DRUG: Proactive TDM-based dosing of secukinumab|DRUG: Proactive TDM-based dosing of ixekizumab|DRUG: Proactive TDM-based dosing of guselkumab	University Hospital, Ghent	PHASE4	INTERVENTIONAL	2024/12/20
Brodalumab	NCT04340076	ACTIVE_NOT_RECRUITING	Psoriasis|Psoriasis Vulgaris	DRUG: Secukinumab|DRUG: Ixekizumab|DRUG: Brodalumab|DRUG: Guselkumab|DRUG: Risankizumab|DRUG: Tildrakizumab|DRUG: Bimekizumab	Radboud University Medical Center	PHASE4	INTERVENTIONAL	2020/8/20
	NCT04306315	ACTIVE_NOT_RECRUITING	Psoriasis Vulgaris	BIOLOGICAL: Brodalumab	LEO Pharma	PHASE4	INTERVENTIONAL	2022/8/16
	NCT04305327	TERMINATED	Psoriasis	DRUG: Brodalumab|DRUG: Ustekinumab|DRUG: Placebo	LEO Pharma	PHASE3	INTERVENTIONAL	2022/12/7
Sonelokimab	NCT07223138	NOT_YET_RECRUITING	Arthritis, Psoriatic	DRUG: Sonelokimab	MoonLake immunotherapeutics AG	PHASE3	INTERVENTIONAL	2025/10/20
	NCT06641076	RECRUITING	Arthritis, Psoriatic	DRUG: Sonelokimab|DRUG: Placebo	MoonLake immunotherapeutics AG	PHASE3	INTERVENTIONAL	2024/10/15
	NCT06641089	RECRUITING	Arthritis, Psoriatic	DRUG: Sonelokimab|DRUG: Placebo|DRUG: Risankizumab	MoonLake immunotherapeutics AG	PHASE3	INTERVENTIONAL	2024/10/15
	NCT05640245	COMPLETED	Arthritis, Psoriatic	DRUG: Sonelokimab|DRUG: Placebo|DRUG: Adalimumab	MoonLake immunotherapeutics AG	PHASE2	INTERVENTIONAL	2022/12/13
SSGJ-608	NCT06242652	ACTIVE_NOT_RECRUITING	Ankylosing Spondylitis	DRUG: 608 Dose A|DRUG: 608 Dose B|DRUG: 608 Dose C|DRUG: Adalimumab|DRUG: Placebo	Sunshine Guojian Pharmaceutical (Shanghai) Co., Ltd.	PHASE2	INTERVENTIONAL	2024/3/19
BAT2306	NCT05377944	COMPLETED	Plaque Psoriasis	DRUG: BAT2306|DRUG: EU-approved Cosentyx	Bio-Thera Solutions	PHASE3	INTERVENTIONAL	2022/10/26
	NCT04711343	COMPLETED	Psoriasis	DRUG: BAT2306|DRUG: Cosentyx (US-licensed)|DRUG: Cosentyx (EU-licensed)	Bio-Thera Solutions	PHASE1	INTERVENTIONAL	2022/6/8
ZL-1102	NCT06380907	RECRUITING	Plaque Psoriasis	DRUG: ZL-1102 1% w/w gel BID for 16 weeks|DRUG: ZL-1102 3% w/w gel BID for 16 weeks|DRUG: ZL-1102 3% w/w gel QD for 16 weeks|DRUG: Placebo ZL-1102 0% w/w gel BID for 16 weeks|DRUG: Placebo ZL-1102 0% w/w gel QD for 16 weeks	Zai Lab (Hong Kong), Ltd.	PHASE2	INTERVENTIONAL	2024/5/22
ORKA-002	NCT06944379	ACTIVE_NOT_RECRUITING	Healthy Volunteers	DRUG: ORKA-002|OTHER: Placebo	Oruka therapeutics, Inc.	PHASE1	INTERVENTIONAL	2025/5/19
Izokibep	NCT05905783	TERMINATED	Hidradenitis Suppurativa	DRUG: Placebo|DRUG: Izokibep	ACELYRIN Inc.	PHASE3	INTERVENTIONAL	2023/6/22
	NCT05623345	TERMINATED	Psoriatic Arthritis	DRUG: Izokibep|DRUG: Placebo to izokibep	ACELYRIN Inc.	PHASE2|PHASE3	INTERVENTIONAL	2022/11/21
	NCT05355805	COMPLETED	Hidradenitis Suppurativa	DRUG: Izokibep|DRUG: Placebo to izokibep	ACELYRIN Inc.	PHASE2	INTERVENTIONAL	2022/5/5
	NCT05384249	TERMINATED	Uveitis	DRUG: Izokibep|DRUG: Placebo	ACELYRIN Inc.	PHASE2	INTERVENTIONAL	2022/8/23
	NCT04706741	COMPLETED	Uveitis	DRUG: Izokibep|DRUG: Prednisone/Prednisolone	ACELYRIN Inc.	PHASE2	INTERVENTIONAL	2022/1/6

The listed conditions represent approved indications or diseases under clinical investigation and should not be interpreted as evidence of established therapeutic efficacy across all disease settings.

### 4.1 Secukinumab

Secukinumab is a fully human IgG1κ monoclonal antibody that selectively binds to IL-17A, preventing its interaction with the IL-17 receptor complex (Gottlieb et al., 2022). By blocking IL-17A-mediated signaling, secukinumab interrupts key pathogenic pathways in psoriasis, restoring immune balance without cross-reactivity to IL-17F or interference with Th1 signaling, which contributes to its favorable safety profile. Structurally, secukinumab forms closed-chain TRICs (trimeric immune complexes), each of which consists of two or more IgG molecules connected by the same number of IL-17A dimers. Moreover, secukinumab, which exhibits the lowest incidence of anti-drug antibodies (ADAs) among IL-17A inhibitors, uniquely forms mainly 2:2 closed-chain complexes (Ungan et al., 2025). Approved by the U.S. FDA in 2015 for moderate-to-severe plaque psoriasis and later for psoriatic arthritis and ankylosing spondylitis, the recommended regimen for psoriasis is 300 mg subcutaneously at weeks 0–4, followed by maintenance dosing every 4 weeks (Kaçar et al., 2022; Megna et al., 2020; Muñoz-Aceituno et al., 2024). Long-term studies, including the SCULPTURE trial, demonstrated sustained efficacy over 5 years, with stable PASI 75/90/100 responses and maintained quality-of-life benefits, without the emergence of new safety concerns (Bissonnette et al., 2018). Furthermore, in the landmark phase 3b MAXIMISE trial, secukinumab (300 mg and 150 mg) demonstrated rapid and significant improvements in axial manifestations of psoriatic arthritis (PsA), achieving substantially higher week-12 ASAS20 responses (63% and 66%, respectively) compared to placebo (31%). These clinical benefits were sustained through 52 weeks and accompanied by objective reductions in spinal and sacroiliac joint inflammation on MRI, maintaining a favorable safety profile (Baraliakos et al., 2021). In addition, secukinumab became the first IL-17A inhibitor approved for HS following successful Phase III trials (SUNSHINE and SUNRISE), demonstrating significant and sustained reduction in inflammatory lesion counts (Kimball et al., 2023). Beyond dermatological and musculoskeletal indications, the therapeutic paradigm of secukinumab has recently extended to large-vessel vasculitis. In the randomized, double-blind, phase II TitAIN trial, secukinumab (300 mg) combined with a glucocorticoid taper regimen achieved a significantly higher sustained remission rate at week 28 compared to placebo (70% vs. 20%) in patients with active giant cell arteritis (GCA), demonstrating favorable tolerability and supporting its development as a viable therapeutic strategy for systemic rheumatic conditions (Venhoff et al., 2023).

### 4.2 Ixekizumab

Ixekizumab is a humanized IgG4 monoclonal antibody that selectively targets IL-17A, preventing its interaction with the IL-17 receptor complex and thereby blocking downstream pro-inflammatory signaling (Liu L. et al., 2016). Structural studies have shown that ixekizumab forms a symmetrical complex with IL-17A, in which two Fab fragments bind to the IL-17A homodimer with two-fold symmetry corresponding to the crystallographic symmetry axis. Its binding interface is contributed almost entirely by a single IL-17A subunit, which explains its lack of cross-reactivity with IL-17F, while still enabling neutralization of the IL-17A/F heterodimer via the A subunit (Ungan et al., 2025). Ixekizumab is produced using recombinant DNA technology in mammalian expression systems and purified through standard bioprocessing methods. Clinical studies, including the IXORA-S and IXORA-R trials, have demonstrated that ixekizumab provides superior clearance of cutaneous and nail lesions compared with ustekinumab and comparable efficacy to guselkumab. Overall, ixekizumab has demonstrated robust efficacy, a favorable safety profile, and durable therapeutic benefit in patients with moderate-to-severe plaque psoriasis. Ixekizumab was approved by the U.S. Food and Drug Administration in March 2016 for the treatment of adult patients with moderate-to-severe plaque psoriasis (Caldarola et al., 2023; Malagoli et al., 2022; Megna et al., 2022).

### 4.3 Bimekizumab

Bimekizumab, a humanized IgG1 monoclonal antibody, was approved by the EMA in 2021 and by the U.S. FDA in 2023 for the treatment of plaque psoriasis, with subsequent regulatory approvals expanding its therapeutic indications to r-axSpA and nr-axSpA (Alinaghi et al., 2019; Oliveira et al., 2021). It selectively inhibits both IL-17A and IL-17F, with binding affinities of 23 pM and 3.2 pM, respectively (Adams et al., 2020). Binding of Bimekizumab Fab to IL-17F is predominantly mediated by the light chain with a buried surface area of 852 Å2 (68.7%) compared with 388 Å2 (31.3%) for the heavy chain. Bimekizumab heavy chain binds to 1 IL-17F monomer, whereas bimekizumab light chain contacts both monomers in the homodimer (Adams et al., 2024). Phase III clinical trials demonstrated rapid onset and durable efficacy with an acceptable safety profile across multiple inflammatory diseases. In the BE VIVID study for plaque psoriasis, 85% of patients achieved PASI90 at week 16 compared with 50% in the ustekinumab group and 4% in the placebo group (Reich et al., 2021a). In BE READY, 91% reached PASI90 at week 16 versus 1% on placeboo (Gordon et al., 2021). The BE RADIANT trial confirmed superior efficacy compared with secukinumab (Reich et al., 2021b). Beyond dermatology, the clinical efficacy of dual IL-17A/IL-17F inhibition has been robustly validated in axial spondyloarthritis through the parallel Phase III trials, BE MOBILE 1 (for nr-axSpA) and BE MOBILE 2 (for r-axSpA). In these studies, bimekizumab treatment yielded high, sustained ASAS40 response rates exceeding 50% at week 52, alongside meaningful improvements in pain, physical function, and objective measures of sacroiliac inflammation. Long-term extension data confirmed the durable suppression of disease activity over 2 years (Baraliakos et al., 2024). The clinical utility of bimekizumab has further expanded into psoriatic arthritis (PsA); in the landmark phase III BE OPTIMAL trial involving biologic-naive patients, bimekizumab (160 mg every 4 weeks) demonstrated rapid, multi-domain efficacy over placebo, yielding significantly higher week-16 ACR50 (44% vs. 10%) and PASI90 (61% vs. 3%) response rates, while effectively suppressing radiographic structural progression and maintaining a well-tolerated long-term safety profile despite a predictable increase in mild-to-moderate oral candidiasis (McInnes et al., 2023). Although oral candidiasis occurred more frequently in patients receiving bimekizumab, the majority of cases were mild and manageable, supporting bimekizumab as a highly effective therapeutic option for both plaque psoriasis and the axial spondyloarthritis spectrum (Freitas and Torres, 2021; Langley et al., 2019).

### 4.4 Brodalumab

Brodalumab is a fully human IgG2 monoclonal antibody that targets and antagonizes the IL-17RA, thereby blocking signaling from multiple IL-17 cytokines, including IL-17A, IL-17C, IL-17E, and IL-17F (Roman and Chiu, 2017). Approved by the U.S. FDA in 2017 for moderate-to-severe plaque psoriasis, it is administered as a 210 mg subcutaneous injection at weeks 0, 1, and 2, followed by maintenance dosing every 2 weeks (Roman and Chiu, 2017). Its efficacy and safety were demonstrated in three pivotal Phase III trials—AMAGINE-1, -2, and -3. In these trials, more than 80% of patients achieved PASI 75, while PASI 100 rates at week 12 were 37%–44% in the brodalumab group versus 19%–22% in the ustekinumab group (Lebwohl et al., 2015; Papp et al., 2016). AMAGINE-1 further confirmed durable benefits over 120 weeks (Papp et al., 2020). Notably, while brodalumab exhibits high efficacy in cutaneous lesions, its clinical application remains strictly limited to psoriasis. Its clinical development for joint diseases, including psoriatic arthritis (PsA) and axial spondyloarthritis, was permanently discontinued. This cessation was driven by critical safety concerns regarding a potential association with suicidal behavior and ideation (Danesh and Kimball, 2016). Consequently, brodalumab was approved in the United States with a Risk Evaluation and Mitigation Strategy (REMS) and carries a black box warning for suicidal ideation and behavior, necessitating careful psychological screening and limiting its broader expansion into therapeutic indications for joint diseases (Lebwohl et al., 2023; Lebwohl et al., 2025).

### 4.5 Vunakizumab

Vunakizumab is a subcutaneous (SC) recombinant humanized anti-IL-17A monoclonal IgG1/κ antibody, being developed by Suzhou Suncadia Biopharmaceutical Co., Ltd. (a subsidiary of Jiangsu Hengrui Pharmaceuticals Co., Ltd.) for the systemic treatment of IL-17 pathway–related autoimmune diseases, including psoriasis, ankylosing spondylitis, and psoriatic arthritis. In August 2024, vunakizumab was approved in China for the treatment of adult patients with moderate-to-severe plaque psoriasis who are eligible for systemic therapy or phototherapy (Keam, 2024). In a phase III clinical trial involving 690 patients randomized in a 2:1 ratio to receive either vunakizumab or placebo, its efficacy was demonstrated to be robust. Patients received 240 mg doses at weeks 0, 2, 4, and 8, followed by maintenance dosing every 4 weeks. Results showed a rapid onset of action, with 56.6% of patients achieving PASI 75 at week 4 and a median time to PASI 90 of 8.3 weeks. PASI 100 responses increased from 36.6% at week 12%–63.1% at week 52. Adverse events were comparable between the treatment and placebo groups, with no new safety concerns, serious infections, or deaths reported (Yan et al., 2025).

### 4.6 Xeligekimab

Xeligekimab is a recombinant human immunoglobulin (Ig)G4 monoclonal antibody that neutralizes IL-17A, being developed by Genrix (Shanghai) Biopharmaceutical for the treatment of plaque psoriasis, axial spondyloarthritis, and lupus nephritis. Xeligekimab binds to IL-17A and blocks its interaction with the IL-17A receptor, thereby inhibiting the release of C-X-C motif chemokine ligand 1 (CXCL1) and IL-6. On 27 August 2024, xeligekimab received approval in China for the treatment of adult patients with moderate-to-severe plaque psoriasis who are candidates for systemic therapy or phototherapy (Blair, 2025). Studies have shown that xeligekimab significantly improved psoriatic lesions after only 2 weeks of treatment. By week 12, the mean PASI improvement reached 91.71%, with PASI 75, PASI 90, and PGA 0/1 response rates of 90.7%, 74.4%, and 74.4%, respectively. At week 52, efficacy continued to improve, with PASI 75 and PASI 90 response rates of 96.5% and 84.1%, respectively, a low relapse rate (0.4%–1.4%), and no evidence of rebound phenomenon observed (Cai et al., 2024). On 14 January 2025, xeligekimab was also approved in China for the treatment of ankylosing spondylitis, becoming the first domestically developed IL-17A inhibitor. In a 48-week, randomized, double-blind, placebo-controlled study conducted across 40 centers in China, xeligekimab demonstrated robust clinical efficacy, with ASAS20 response rates of 74.0% and 65.8% in the 200 mg and 100 mg groups, respectively, compared with 35.9% in the placebo group (P < 0.001) (Zhang et al., 2025).

### 4.7 Netakimab

Netakimab is an IL-17A–specific monoclonal antibody developed by Biocad and was approved in Russia in 2019 for the treatment of moderate-to-severe plaque psoriasis, psoriatic arthritis, and ankylosing spondylitis. The antibody comprises a Lama glama–derived VHH domain and a variable light (VL) domain. Previous studies have determined the crystal structure of the IL-17A-specific VHH domain in complex with IL-17A at 2.85 Å resolution. Specific amino acid residues within the three complementarity-determining regions (CDRs) of the VHH domain form a network of solvent-inaccessible hydrogen bonds with two epitope regions on IL-17A (Kostareva et al., 2022). In a randomized, double-blind controlled trial, 213 patients with moderate-to-severe plaque psoriasis were randomly assigned to receive NTK at a dose of 120 mg once every 2 weeks (NTK Q2W), NTK at a dose of 120 mg once every 4 weeks (NTK Q4W), or placebo. At week 12, 77.7%, 83.3%, and 0% of patients in the NTK Q2W, NTK Q4W, and placebo groups, respectively, achieved a PASI 75 response [(P < 0.0001, Fisher’s exact test) ITT]. The effect was maintained throughout the 1-year treatment. NTK showed a good safety profile and low immunogenicity (Puig et al., 2021).

### 4.8 Other IL-17-targeted therapeutic modalities

The therapeutic landscape for IL-17-mediated inflammatory diseases is rapidly evolving beyond conventional monoclonal antibodies (mAbs) toward a diverse array of engineered molecular architectures. Currently, the clinical pipeline is characterized by a dual-track progression: the refinement of conventional IgG frameworks and the emergence of non-canonical protein scaffolds designed to overcome pharmacological bottlenecks such as suboptimal tissue bioavailability and limited multi-pathway intervention.

While established biologics continue to define the standard of care, several optimized mAbs and biosimilars have entered pivotal clinical stages. SSGJ-608 is a humanized anti-IL-17A monoclonal antibody independently developed by the Chinese company Sinocelltech, featuring a novel amino acid sequence. It selectively binds to and neutralizes IL-17A, inhibiting the release of pro-inflammatory factors and effectively alleviating psoriatic inflammation. Phase III clinical trials demonstrated that all primary (PASI 75, sPGA 0/1) and key secondary (PASI 90, PASI 100, sPGA 0) efficacy endpoints were successfully met. SSGJ-608 has been submitted for regulatory approval for the treatment of adult patients with moderate-to-severe plaque psoriasis. Similarly, BAT2306 is an IgG1κ monoclonal antibody targeting human IL-17A and serves as a biosimilar of secukinumab. By selectively binding IL-17A and blocking its interaction with the receptor, it inhibits the release of pro-inflammatory cytokines and chemokines. The marketing application for BAT2306 has been accepted by the Chinese NMPA, and preparations are underway for submission to the FDA and EMA. Furthermore, several IL-17A/IL-17F-targeting monoclonal antibodies have entered pivotal clinical stages, creating a competitive head-to-head developmental landscape. In Phase III trials, CT-P55 (Celltrion), CMAB-015 (Maybridge Pharma), SYS-6012 (CSPC), HB-0017 (Huaao Biopharma), QX002N (Quxin Biotech), and Roconkibart (Rui Shi Biotech) have completed or are near completion of patient enrollment, with some reporting interim results demonstrating efficacy and safety comparable to approved reference products.

To address the redundancy of IL-17-mediated signaling, multispecific platforms have emerged as a pivotal strategy. Sonelokimab is a trivalent humanized nanobody targeting IL-17A and IL-17F, incorporating an albumin-binding domain for extended half-life. Its small size enables superior tissue penetration and enhanced tissue distribution, and it shows more potent inhibition of IL-17 signaling compared with secukinumab. Phase I data demonstrated an acceptable safety profile, dose-dependent clinical improvement, an elimination half-life of 11–12 days, and marked reductions in psoriasis-associated gene expression (McInnes et al., 2025; Papp et al., 2021). Similarly, LZM012, a China-developed dual-target humanized IgG1 monoclonal antibody against IL-17A and IL-17F, inhibits inflammatory pathways by blocking IL-17A/F receptor binding. In Phase III psoriasis studies, it achieved a PASI 100 response rate of 49.5% at week 12 and a PASI 75 response rate of 65.7% at week 4, demonstrating promising clinical efficacy. More recently, Oruka Therapeutics announced that its novel dual-target IL-17A and IL-17F antibody ORKA-002 has initiated dosing in healthy volunteers in a Phase I clinical trial, with interim data expected by the end of 2025 and Phase II development planned for early 2026. ORKA-002’s dual-target mechanism and extended half-life may allow only 2–3 doses per year, potentially offering a competitive therapeutic option for psoriasis and other inflammatory skin diseases. Bispecific antibodies (bsAbs) have emerged as a compelling strategy to address the redundancy and complexity of IL-17–mediated signaling. Mechanistic insights from minimal physiologically based pharmacokinetic (mPBPK) modeling indicate that simultaneous neutralization of IL-17A and IL-17F can produce synergistic anti-inflammatory effects while enhancing drug distribution within inflamed tissues (Spinosa et al., 2026). Notably, fine-tuning the binding affinities of each arm enables optimization of target engagement and minimizes off-target interactions. Recently, a fully synthetic nanobody library was utilized to develop a novel bispecific nanobody, designated as BiNB, which simultaneously targets B-cell activating factor (BAFF) and interleukin-17 (IL-17). Structurally, BiNB was engineered by linking two screened inhibitory single-domain antibodies—NB1 (anti-BAFF) and NB2 (anti-IL-17)—via a flexible linker. Pharmacologically, this dual-targeting construct (BiNB) effectively antagonizes BAFF-dependent B-cell proliferation and IL-17-driven CXCL1 secretion without mutual interference, offering a synergistic therapeutic candidate for complex autoimmune disorders (Xin et al., 2025).

In the targeted IL-17 treatment strategy, in addition to traditional monoclonal antibodies, various new intervention methods have gradually attracted attention in recent years, mainly including affibodies, DARPins, and other non-conventional architectures. Izokibep, an IL-17A–specific Affibody, exhibits approximately 1000-fold higher binding affinity than conventional monoclonal antibodies while maintaining a substantially reduced molecular size (∼1/8 of an IgG), thereby facilitating improved tissue penetration. Clinical evidence from a phase II trial (n = 135) supports its favorable efficacy and safety profile (Behrens et al., 2022), with further evaluation in spondyloarthritis ongoing. In parallel, Zai Lab initiated a global Phase II study evaluating its IL-17 inhibitor ZL-1102 for chronic plaque psoriasis. ZL-1102 is a fully human VH antibody fragment targeting IL-17A, innovatively developed for mild-to-moderate psoriasis, distinct from systemic IL-17 inhibitors.

Advances in antibody engineering have further enabled multifunctional formats such as Fab–scFv fusion proteins have demonstrated improved localization and retention within inflamed tissues, partly mediated by neonatal Fc receptor (hFcRn)–dependent transport. This strategy has shown promising anti-inflammatory effects in *ex vivo* human inflammatory bowel disease biopsies, overcoming the limited tissue penetrability often observed with conventional biologics (He et al., 2025). Complementing these protein-based strategies, vaccination strategies targeting IL-17A have emerged as a promising approach to elicit endogenous neutralizing antibodies. For instance, a virus-like particle–based vaccine (Qβ-IL-17) has demonstrated significant attenuation of disease severity in preclinical models of autoimmune disorders (Dallenbach et al., 2015), highlighting its potential as a long-acting immunotherapeutic modality. Beyond vaccination, alternative protein scaffolds such as Affibody molecules have gained increasing attention. In parallel, peptide-based inhibitors represent another innovative class of IL-17–targeting therapeutics. Linear peptides, exemplified by the 15-residue high-affinity peptide (HAP), function as competitive antagonists by symmetrically engaging the IL-17A homodimer in a 2:1 stoichiometry. Structurally, these peptides insert their N-terminal segments into the interfacial region between IL-17A subunits, thereby inducing steric hindrance that disrupts IL-17A/IL-17RA interaction and suppresses downstream pro-inflammatory signaling (Liu S. et al., 2016). Collectively, these diverse modalities underscore a paradigm shift toward site-specific enrichment and multi-target precision in IL-17 therapeutics.

## 5 Small molecule inhibitors

**FIGURE 3:**
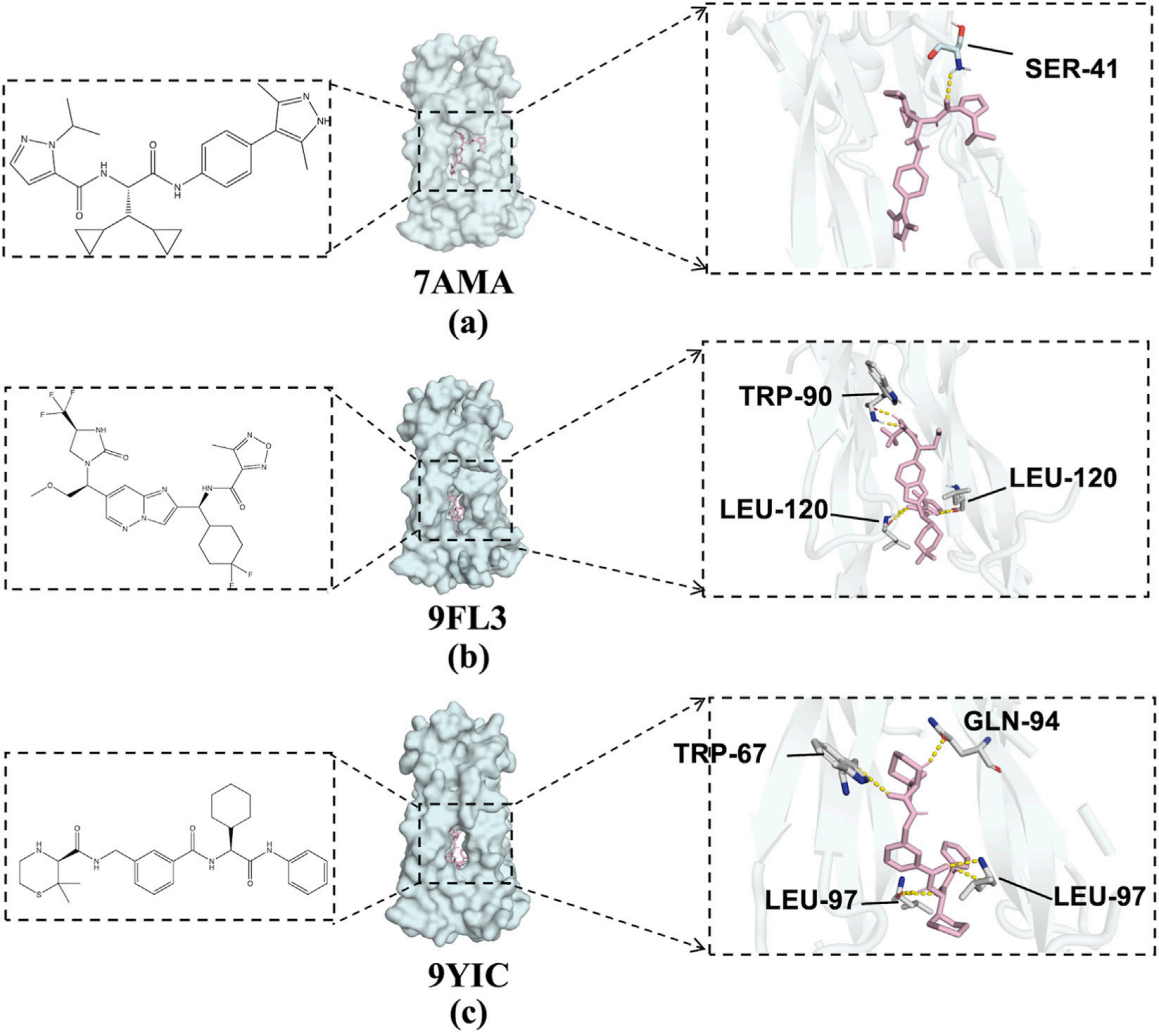
Molecular docking and binding mode analysis of IL-17 in complex with small-molecule inhibitors. The panels illustrate the chemical structures (left), 3D surface docking poses (middle), and detailed intermolecular interactions (right) within the binding pockets. **(a)** Docking complex of IL-17 with LEO 153339, highlighting a key hydrogen bond interaction with the amino acid residue SER-41. **(b)** Docking complex of IL-17 with LY3509754, demonstrating critical binding interactions with TRP-90 and LEU-120 residues. **(c)** Docking complex of IL-17 with JNJ627, exhibiting specific network interactions with TRP-67, GLN-94, and LEU-97 residues.

In recent years, growing attention has been directed toward the development of small-molecule inhibitors targeting the IL-17 signaling pathway (Figure 3), which offer distinct advantages in oral bioavailability, tissue penetration, and modulation of IL-17 signaling. Collectively, these developments highlight the increasing therapeutic potential of small-molecule IL-17 inhibitors as complementary or alternative therapeutic strategies, as summarized in Table 3.

**TABLE 3:** Small molecule inhibitors of the IL-17 pathway.

Drug	NCT number	Study status	Conditions	Interventions	Sponsor	Phases	Study type	Start date
Xyngari (DMT-310)	NCT06090721	COMPLETED	Acne Vulgaris	DRUG: DMT310|DRUG: Placebo	Dermata Therapeutics	PHASE3	INTERVENTIONAL	2023/12/8
	NCT04106778	COMPLETED	Acne Vulgaris	DRUG: DMT310|DRUG: Placebo	Dermata Therapeutics	PHASE2	INTERVENTIONAL	2019/10/10
	NCT03536637	COMPLETED	Acne Vulgaris	DRUG: DMT310|DRUG: Hydrogen Peroxide|DRUG: Placebo	Dermata Therapeutics	PHASE2	INTERVENTIONAL	2018/5/22
	NCT05108025	UNKNOWN	Acne Rosacea	DRUG: Topical Powder|DRUG: Placebo topical Powder	Dermata Therapeutics	PHASE2	INTERVENTIONAL	2021/11/15
Simepdekinra (DC-853/LY4100511)	NCT06311656	COMPLETED	Healthy	DRUG: LY4100511 (DC-853)|DRUG: LY4100511 (DC-853)|DRUG: LY4100511 (DC-853)|DRUG: LY4100511 (DC-853)|DRUG: Placebo	DICE Therapeutics, Inc., a wholly owned subsidiary of Eli Lilly and Company	PHASE1	INTERVENTIONAL	2024/3/19
	NCT06937411	COMPLETED	Healthy	DRUG: LY4100511	Eli Lilly and Company	PHASE1	INTERVENTIONAL	2023/2/13
	NCT06916143	RECRUITING	Healthy Participants	DRUG: LY4100511|DRUG: Rabeprazole	Eli Lilly and Company	PHASE1	INTERVENTIONAL	2025/4/8
	NCT06345794	COMPLETED	Healthy	DRUG: LY4100511 (DC-853)|DRUG: Itraconazole|DRUG: Fluconazole|DRUG: Carbamazepine	DICE Therapeutics, Inc., a wholly owned subsidiary of Eli Lilly and Company	PHASE1	INTERVENTIONAL	2024/4/3
	NCT06627088	COMPLETED	Healthy	DRUG: LY4100511 (DC-853)|DRUG: [14C]-LY4100511 (DC-853) Administered oral dose|DRUG: LY4100511 (DC-853)|DRUG: [14C]-LY4100511 (DC-853)	DICE Therapeutics, Inc., a wholly owned subsidiary of Eli Lilly and Company	PHASE1	INTERVENTIONAL	2024/8/23
​	NCT06503679	COMPLETED	Healthy	DRUG: LY4100511 (DC-853) Dose 1|DRUG: LY4100511 (DC-853) Dose 2|DRUG: LY4100511 (DC-853) Dose 3|DRUG: Midazolam|DRUG: Repaglinide|DRUG: Digoxin|DRUG: Rosuvastatin	DICE Therapeutics, Inc., a wholly owned subsidiary of Eli Lilly and Company	PHASE1	INTERVENTIONAL	2024/7/16
​	NCT06602219	COMPLETED	Plaque Psoriasis	DRUG: LY4100511|DRUG: Placebo	DICE Therapeutics, Inc., a wholly owned subsidiary of Eli Lilly and Company	PHASE2	INTERVENTIONAL	2024/10/25
LEO 153339	NCT04883333	COMPLETED	Healthy Volunteers	DRUG: LEO 153339|DRUG: Placebo	LEO Pharma	PHASE1	INTERVENTIONAL	2021/5/17
UA026	NCT07038720	RECRUITING	Plaque Psoriasis|Healthy Volunteer	DRUG: UA026|DRUG: Placebo	Usynova Pharmaceuticals Ltd.	PHASE1	INTERVENTIONAL	2025/5/8
LY3509754	NCT04586920	TERMINATED	Healthy	DRUG: LY3509754|DRUG: Placebo|DRUG: Itraconazole|DRUG: Midazolam	Eli Lilly and Company	PHASE1	INTERVENTIONAL	2020/10/20
	NCT04152382	TERMINATED	Psoriasis	DRUG: LY3462817 - IV|DRUG: LY3462817 - SC|DRUG: Placebo - IV|DRUG: Placebo - SC|DRUG: LY3509754|DRUG: Placebo	Eli Lilly and Company	PHASE1	INTERVENTIONAL	2019/11/20

### 5.1 LEO 153339

**FIGURE 4:**
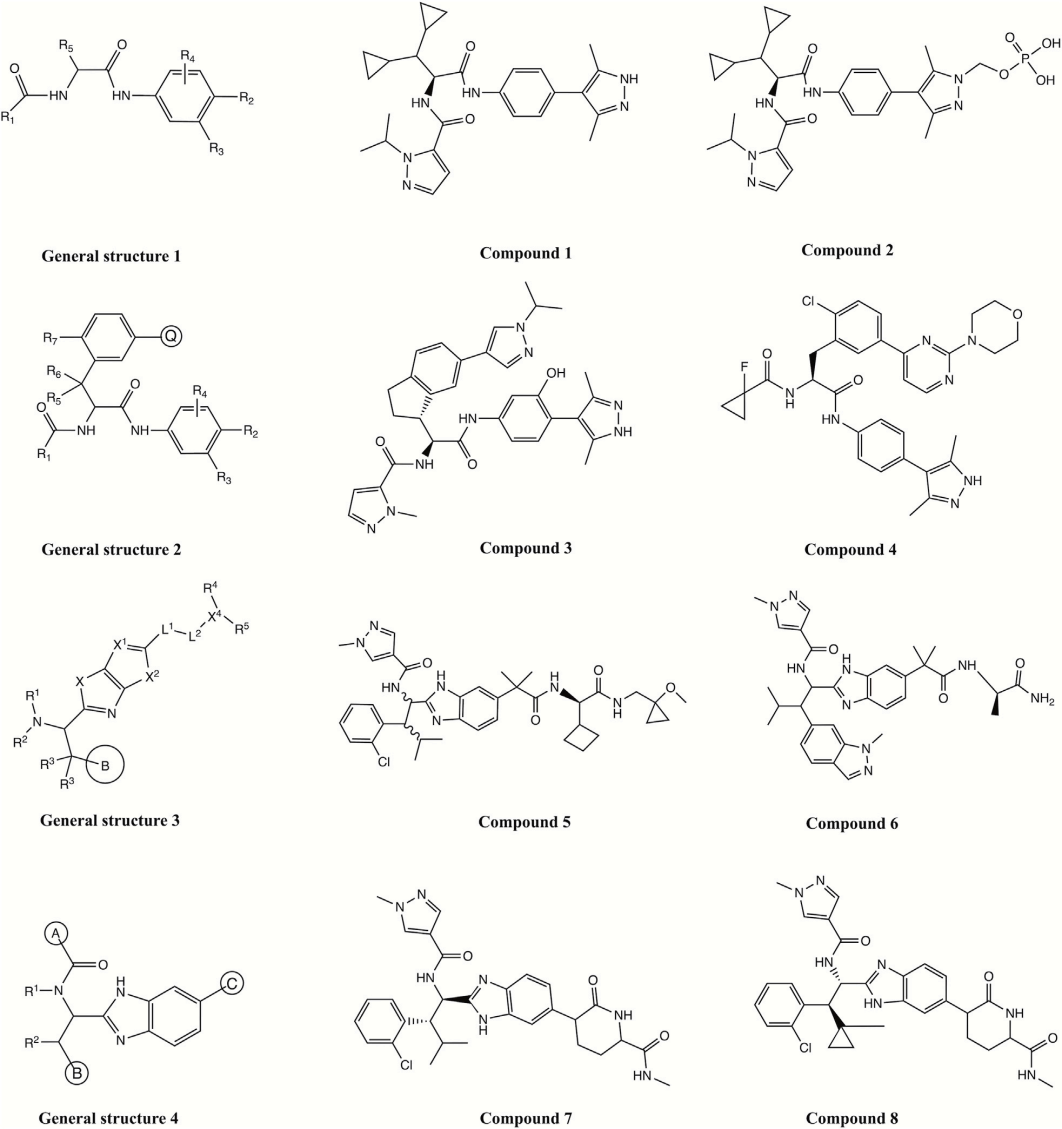
Chemical skeletons and representative structures of small-molecule IL-17 inhibitors.

LEO 153339 (developed by LEO Pharma) is an orally bioavailable small molecule that acts as a direct inhibitor of the IL-17A/IL-17RA axis. Unlike broader immunomodulators that affect upstream signaling or multiple cytokines, LEO 153339 and its related series (amino-acid anilides and derivatives, general structures 1 and 2 in Figure 4) specifically target the PPI between the IL-17A cytokine and its receptor IL-17RA. Published in 2020, these compounds represent a significant advancement in direct IL-17A modulation. Within this series, the most potent variants, including compounds 1, 2, 3, and 4, exhibit exceptional inhibitory activity with nanomolar EC_50_ values in functional assays. Notably, compound 1 demonstrated favorable oral bioavailability in preclinical rodent and non-rodent pharmacokinetic studies, while compound 2, acting as a prodrug of compound 1, displayed a superior *in vivo* pharmacokinetic profile. The structure-activity relationship (SAR) governing this direct inhibition is summarized in Figure 3a. From a clinical application perspective, LEO 153339 holds significant potential as a convenient, oral alternative to current injectable anti-IL-17 monoclonal antibodies for managing chronic inflammatory and autoimmune disorders. However, transitioning this molecule from bench to bedside faces distinct pharmacological hurdles. While highly effective against IL-17A homodimers, LEO 153339 exhibits limited potency against IL-17A/F heterodimers, which may constrain its clinical efficacy in diseases driven synergistically by both isoforms. Additionally, the complex metabolic conversion required by its prodrug design, along with potential off-target safety risks in humans, necessitates rigorous clinical translational validation. Future optimization must balance systemic safety with oral potency to fully unlock its therapeutic value.

### 5.2 HGP0508

In 2019, HitGen Inc. disclosed a series of imidazole derivatives, designated as general structure 3, which function as direct inhibitors of the IL-17A/IL-17RA axis. These compounds achieve their therapeutic effect by specifically disrupting the PPI between the IL-17A cytokine and its receptor, with exemplary potent variants exhibiting nanomolar IC_50_ values. Subsequently, in 2021, HitGen expanded this chemical space with a class of benzimidazole-based derivatives, classified as general structure 4. Although certain representative examples in this second series, such as compounds 7 and 8, showed more moderate micromolar to millimolar potency, they remain categorized as direct IL-17A modulators based on their targeted binding mechanism at the IL-17A/IL-17RA interface rather than indirect systemic immune regulation. The scaffold evolution of these direct inhibitors is illustrated in Figure 4. The primary clinical application of HGP0508 lies in its potential to provide a non-biologic, orally accessible therapeutic option for chronic inflammatory diseases, bypassing the immunogenicity and administration constraints associated with monoclonal antibodies. However, advancing these compounds into clinical translation presents notable pharmacological and druggability challenges. The structural complexity of their multi-substituted heterocyclic cores often introduces physicochemical hurdles, such as high molecular weight and sub-optimal solubility, which can hinder *in vivo* bioavailability. Furthermore, for future clinical viability, structural optimization must transition from merely enhancing binding affinity to prioritizing high selectivity across the broader IL-17 cytokine family, thereby mitigating potential off-target toxicities during long-term chronic treatment.

### 5.3 Simepdekinra (DC-853/LY4100511)

**FIGURE 5:**
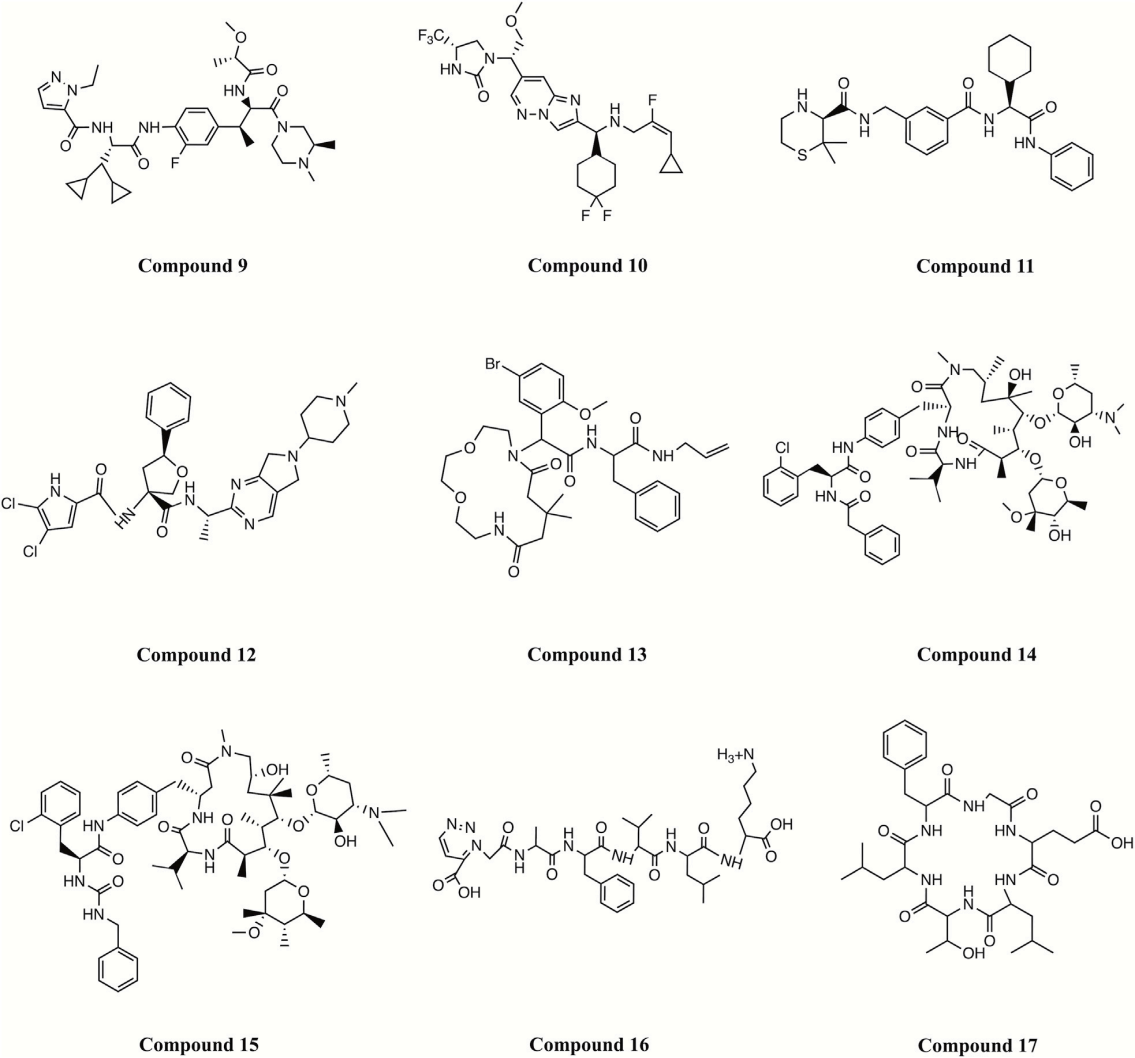
Chemical structures and representative profiles of small-molecule and macrocyclic IL-17 modulators.

Simepdekinra (Compound 9), developed by DICE Therapeutics (now Eli Lilly), is a potent, orally bioavailable direct IL-17A inhibitor. It functions by physically blocking the PPI interface between the IL-17A cytokine and the IL-17RA receptor, thereby preventing the initiation of downstream pro-inflammatory signaling. In functional assays, Simepdekinra demonstrated exceptional direct inhibitory activity, exhibiting nanomolar affinity against both IL-17A/A homodimers and IL-17A/F heterodimers (Figure 5). Due to its high specificity and direct mode of action, Simepdekinra is currently being investigated for the clinical treatment of IL-17A-mediated inflammatory diseases, such as psoriasis, ankylosing spondylitis, and psoriatic arthritis. From a pharmacological perspective, the clinical translation of Simepdekinra underscores the unique challenges of targeting the traditionally “undruggable” IL-17A/IL-17RA PPI interface. Because IL-17A presents a large, relatively flat dimeric surface lacking deep hydrophobic pockets, small molecules like Compound 9 require sophisticated structure-based designs to induce functional binding cavities. This structural constraint directly impacts its biopharmaceutical properties. In preclinical non-human primate models, Simepdekinra exhibited suboptimal absolute oral bioavailability, lower drug exposure, and higher clearance rates compared to next-generation candidates, indicating that balancing ADME properties with high-affinity PPI disruption remains a primary hurdle. Future development must focus on structural optimization to enhance systemic exposure and sustain clinical efficacy.

### 5.4 LY3509754

LY3509754 is a potent, orally bioavailable small molecule that functions by specifically binding to the IL-17A cytokine, thereby directly disrupting its PPI with the IL-17RA receptor. The SAR governing this direct binding scaffold are shown in Figure 3b. Although its direct anti-inflammatory mechanism demonstrated high biological efficacy, the clinical development of LY3509754 was discontinued during Phase 1 trials due to unexpected systemic toxicities. Subsequent optimization studies investigated whether modifying its core chemical scaffold could mitigate these toxicological risks while maintaining target potency (Velcicky et al., 2024). Through various structural modifications, including the development of optimized variants such as compound 10 (Figure 5), researchers successfully sustained robust anti-inflammatory efficacy, which effectively reduced knee joint swelling in preclinical rat arthritis models. However, subsequent toxicological profiling revealed that adverse findings persisted despite these scaffold modifications. From a clinical and translational perspective, the failure of LY3509754 underscores a critical challenge in small-molecule PPI inhibitor development: the decoupling of potent target efficacy from systemic safety. The persistent toxicity observed even after structural optimization (such as in compound 10) suggests that the adverse effects might stem from off-target interactions inherent to the underlying chemical chemotype, or potentially from a narrow therapeutic window associated with systemic IL-17A blockade. Consequently, the clinical application of such direct inhibitors requires a strategic shift. Future development must prioritize druggability and safety by implementing more rigorous, early-stage toxicological profiling and exploring tissue-specific delivery strategies to mitigate systemic risks while preserving the potent direct inhibition mechanism.

### 5.5 JNJ627

Recently, a novel class of inhibitors targeting the IL-17A signaling axis was identified utilizing DNA-encoded library (DEL) technology (Milla et al., 2025). The lead molecule of this series, JNJ627 (Compound 11) (Figure 5), binds within the internal interface of the IL-17A homodimer, thereby preventing its engagement with the cognate receptor. Instead of competing at the canonical receptor-binding site, these compounds exert their activity by allosterically perturbing the quaternary structure of IL-17A, blocking the conformational arrangement required for receptor recognition (Figure 3c). Notably, this chemotype displays exceptionally slow dissociation kinetics and robustly suppresses IL-17A–driven responses in human primary cells. From a clinical application and druggability perspective, JNJ627’s unique allosteric mechanism offers a potential therapeutic advantage by achieving prolonged target engagement. However, a major translational challenge is determining whether this biochemical binding persistence can successfully compensate for the transient systemic exposure typical of small molecules compared to long-acting biologics. Furthermore, the structural complexity inherent in DEL-derived hits often requires extensive chemical optimization to balance potent cellular activity with favorable ADME properties, particularly in ensuring consistent oral absorption for chronic inflammatory conditions. Future evaluation should focus on whether this specific allosteric site provides superior selectivity over other IL-17 isoforms to minimize systemic off-target risks while sustaining robust clinical efficacy.

### 5.6 AbbVie series

Recent work has described a novel class of small-molecule IL-17A inhibitors discovered via DNA-encoded chemical library screening and optimized for *in vivo* efficacy (Ramos et al., 2024). Notably, these PPI inhibitors engage IL-17A through a distinctive binding mode, wherein two ligand molecules symmetrically occupy the central cavities of the IL-17A homodimer (Figure 5). This optimized structural series demonstrated low-nanomolar potency and oral efficacy in preclinical models, highlighting its potential as an orally bioavailable alternative to current antibody-based IL-17A therapies. From a clinical application and translational perspective, the clinical utility of this novel 2:1 binding stoichiometry introduces a unique pharmacological challenge, as it requires simultaneous occupancy of both binding pockets to ensure stable and sustained IL-17A inhibition under dynamic *in vivo* physiological conditions. Furthermore, from a druggability standpoint, although chemical optimization successfully enhanced metabolic stability, the relatively high molecular weight and lipophilicity inherent in this symmetrical scaffold may constrain long-term human safety and oral bioavailability compared to traditional small-molecule therapies. Future clinical validation must also critically evaluate whether this symmetric binding mode provides sufficient cross-reactivity and selectivity against IL-17A/F heterodimers, which is essential for achieving therapeutic parity with established pan-IL-17 biologics in diverse inflammatory contexts.

### 5.7 UA026

UA026 (developed by Yousen Jianheng Biopharmaceutical) is an orally administered small-molecule IL-17A inhibitor and the first domestic candidate in its class within China to enter clinical development. UA026 functions by directly blocking the binding of IL-17A to its cognate receptor, thereby suppressing downstream pro-inflammatory signaling cascades and downstream inflammatory mediators to attenuate cutaneous lesions and alleviate the clinical manifestations of psoriasis. Following promising preclinical profiles demonstrating robust potency, selectivity, and a favorable safety margin, this candidate has successfully advanced into Phase 1 clinical evaluation, marking a significant milestone in oral IL-17A targeted translation. From a clinical application standpoint, UA026 offers a promising non-biologic alternative designed to bypass the stability and administration constraints of injectable monoclonal antibodies. However, its ultimate clinical success will largely depend on its ability to demonstrate non-inferiority in Psoriasis Area and Severity Index (PASI) response rates compared to global frontrunners like Simepdekinra and established biological therapies. Furthermore, given the chronic nature of psoriasis management, future clinical investigations must critically address long-term druggability challenges, ensuring that its preclinical safety translates into a lack of cumulative systemic toxicity or gastrointestinal intolerance during extended human exposure. Incorporating robust pharmacodynamic biomarkers in upcoming clinical phases will be essential to accurately bridge the gap between early-stage dosing and late-stage therapeutic efficacy.

### 5.8 Macrocyclic modifiers

Macrocyclic compounds represent an emerging class of IL-17A inhibitors designed to overcome the limitations of conventional linear small molecules in targeting flat PPI interfaces. A recent study reported the discovery of macrocyclic IL-17A modifiers identified through an integrated workflow combining pharmacophore modeling and virtual screening (Abdelraheem et al., 2022). Structurally, these macrocycles utilize a constrained conformational scaffold that forms key hydrogen bonds and hydrophobic contacts at the dimer interface, a pattern consistent with established IL-17A antagonists. Unlike conventional linear inhibitors, these macrocyclic agents bind at the interface of the IL-17A homodimer, thereby allosterically disrupting its interaction with the IL-17 receptor. Utilizing a targeted virtual library, candidate molecules were synthesized, among which Compound 13 and its stereoisomers exhibited the most promising binding affinities, demonstrating nanomolar dissociation constants (Kd) (Figure 5). From a clinical application and druggability perspective, macrocyclic modifiers offer a distinct therapeutic strategy, combining the high affinity and selectivity typical of biologics with the potential stability of non-antibody treatments. However, transitioning these candidates into clinical translation poses significant pharmacological challenges. The structural complexity and inherently high polar surface area of these macrocyclic frameworks can severely limit oral absorption and overall bioavailability. Future investigations must prioritize evaluating the translational potential of these candidates by shifting focus from cell-free biochemical affinity to robust *in vivo* pharmacokinetic validation, while ensuring that this scaffold sustains sufficient selectivity across broader IL-17 family isoforms to prevent off-target risks.

### 5.9 Macrocycle series

To target the challenging IL-17A/IL-17RA interface, researchers utilized a macrolide-inspired macrocycle platform to identify a series of candidates with high therapeutic potential (Koštrun et al., 2021). Within this series, Compound 14 was identified as a representative macrocyclic lead from the initial optimization phase, showing significant binding affinity and effectively inhibiting downstream IL-8 secretion in primary human cells (Figure 5). Through SAR refinements focusing on the macrocyclic attachment points and side-chain terminals, researchers developed a superior derivative, Compound 15. Compared to its predecessor, Compound 15 maintains potent nanomolar inhibitory activity while demonstrating significantly optimized *in vivo* physicochemical properties, notably overcoming the absorption barriers typical of PPI small molecules to achieve moderate oral bioavailability. From a clinical application perspective, this series underscores that mimicking the constrained conformational characteristics of natural macrocycles can effectively block the protein contact interfaces within the central region of the IL-17A homodimer, offering a promising oral alternative to injectable monoclonal antibodies for autoimmune diseases. However, the clinical translation of these macrocyclic candidates faces distinct druggability hurdles, particularly regarding the trade-off between molecular weight and passive membrane permeability. The high conformational flexibility required for these large scaffolds to shield polar groups during membrane transit may lead to unpredictable metabolic stability and systemic clearance in humans. Furthermore, future investigations must evaluate whether this increased structural complexity provides a sufficient selectivity margin across the broader IL-17 cytokine family to justify the complex synthetic scale-up processes compared to traditional small-molecule inhibitors.

### 5.10 IL-17RB/MLK4 cyclic peptide modulators

A novel class of IL-17 pathway–targeting agents has been developed based on structure-guided cyclic peptides designed to disrupt PPIs within the IL-17 signaling axis (Hu et al., 2025). Specifically, these cyclic peptide candidates target the IL-17RB–MLK4 interaction, a critical oncogenic signaling node implicated in cancer progression. Engineered from the loop region of the IL-17RB SEFIR domain, these agents utilize conformational cyclization to significantly improve structural stability, cellular uptake, and intracellular persistence compared to their linear precursors. Functionally, these macrocyclic candidates demonstrate robust antitumor and anti-metastatic efficacy in preclinical functional and evaluation assays. Mechanistically, the cyclic peptide functions as a specific PPI disruptor by directly binding to the kinase domain of MLK4, thereby preventing its association with IL-17RB. This physical disruption blocks IL-17B–induced downstream oncogenic signaling cascades without suppressing the intrinsic kinase activity of MLK4, illustrating a highly selective pharmacological profile driven by topological and conformational stability. From a clinical application standpoint, targeting the non-canonical IL-17RB/MLK4 axis with these cyclic peptides offers a promising, highly selective targeted therapeutic strategy for aggressive malignancies, such as pancreatic cancer, which are often refractory to conventional therapies. However, transitioning these peptide candidates into clinical practice faces distinct druggability and translational bottlenecks. Despite the structural gains from cyclization, the therapeutic utility of these scaffolds remains constrained by inherent pharmacokinetic limitations, including susceptibility to proteolytic degradation and a limited systemic half-life in human subjects. To enhance future clinical viability, structural optimizations must focus on exploring non-natural amino acid substitutions or integrating advanced drug delivery systems to effectively breach the complex physiological barriers of the tumor microenvironment, ensuring that potent preclinical PPI disruption translates into durable clinical outcomes.

### 5.11 Pharmacophore-derived candidates

A recent study employing ligand-based pharmacophore modeling combined with virtual screening and molecular dynamics (MD) simulations identified a novel series of IL-17A inhibitors, among which compound 16 and compound 17 emerged as representative candidates from peptide and macrocyclic classes, respectively (Figure 5) (Razzaq et al., 2026). Structurally, compound 16 functions as a flexible, linear peptide-like molecule that optimizes receptor engagement through extensive hydrogen bonding within the IL-17A interface. In contrast, compound 17 utilizes a macrocyclic framework whose conformational rigidity facilitates a well-defined and highly stable binding orientation within the central pocket of the IL-17A homodimer. Mechanistically, both candidates achieve their therapeutic potential by specifically occupying the large, shallow PPI interface of IL-17A, utilizing a complementary combination of polar contacts and hydrophobic interactions to allosterically disrupt downstream receptor recognition. From a clinical application and translational perspective, these pharmacophore-derived candidates offer valuable structural archetypes for non-antibody alternatives in treating chronic inflammatory disorders. However, transitioning these computational hits into clinical utility faces substantial druggability barriers. In silico evaluations indicate that their relatively high molecular weight and high topological polar surface area (TPSA)—typical of small-molecule PPI binders—may severely limit passive membrane permeability and intestinal absorption. Because explicit experimental clearance or absolute oral bioavailability data remain unavailable, their predicted low systemic toxicity profiles require rigorous translational confirmation. Future research must prioritize robust *in vitro* and *in vivo* experimental validation to determine whether the exceptional binding stability demonstrated in computational models can successfully overcome permeability limitations and achieve therapeutic concentrations in living systems.

### 5.12 Natural-derived compounds

Although this review primarily focuses on synthetic small-molecule inhibitors, several natural product-derived or nature-inspired agents have also been reported to modulate the IL-17 pathway. For instance, cycloketoboswellic acid (CKBA), the active component of SIM0335, suppresses IL-17A expression by regulating Th17 cell differentiation via inhibition of *de novo* fatty acid synthesis, while Xyngari (DMT-310), a sponge-derived topical formulation, exerts anti-inflammatory effects through both mechanical and bioactive chemical components. In addition, plant-derived compounds such as curcumin, resveratrol, andrographolide, and berberine have been widely reported to regulate Th17 responses and IL-17 signaling in various inflammatory conditions. However, given the structural diversity and distinct development paradigms of such agents, natural product-derived modulators are not discussed in detail in this review.

## 6 Conclusion

In conclusion, IL-17 is a central mediator in the pathogenesis of a broad range of inflammatory, autoimmune, and tumor-associated diseases, highlighting the IL-17 signaling axis as an important therapeutic target. Increasing evidence demonstrates extensive crosstalk between IL-17 and other inflammatory pathways, particularly the IL-23/IL-17 axis, which plays a pivotal role in Th17 cell differentiation and amplification of inflammatory responses. These findings not only deepen our understanding of disease mechanisms but also support the development of multi-target therapeutic strategies to achieve improved clinical efficacy. Driven by these mechanistic insights, substantial progress has been made in IL-17-targeted drug development. Monoclonal antibodies, including secukinumab, ixekizumab, and brodalumab, have shown significant efficacy in psoriasis and related inflammatory disorders, validating IL-17 as a clinically actionable target. In parallel, several next-generation therapeutics remain under clinical investigation. Beyond conventional antibodies, emerging modalities such as nanobodies, antibody fragments, DARPins, phosphopeptides, and multispecific constructs are further expanding the therapeutic landscape and providing new opportunities for precision medicine. Alongside biologics, small-molecule inhibitors have attracted increasing attention because of their potential advantages, including oral administration, lower manufacturing costs, improved tissue penetration, and greater flexibility for structural optimization. However, the development of IL-17 small-molecule inhibitors remains particularly challenging due to the shallow and dynamic nature of the IL-17 protein–protein interaction interface. Most currently reported compounds are based on highly convergent bis-amide scaffolds that target the IL-17A dimer interface, thereby limiting chemical diversity and opportunities for pharmacokinetic optimization. Consequently, recent drug-design strategies have increasingly shifted toward targeting IL-17 ligands, IL-17 receptors, or the ligand–receptor interface through structure-guided and conformationally driven approaches. Within this context, macrocyclic inhibitors have emerged as promising “middle-space” therapeutics bridging conventional small molecules and biologics. Their conformationally constrained architectures can improve binding affinity, selectivity, and physicochemical properties while maintaining opportunities for further structural optimization. Despite these advances, several challenges remain, including balancing potency with oral bioavailability, improving *in vivo* translational efficacy, overcoming pathway redundancy, and minimizing long-term safety risks. Overall, continuing advances in medicinal chemistry, structural biology, and protein engineering are accelerating the evolution of IL-17-targeted therapeutics from conventional neutralizing antibodies toward more diverse and multifunctional platforms. Future research should focus on integrating biologic- and small-molecule-based strategies, optimizing efficacy and safety profiles, and developing multi-target or multifunctional therapeutics to achieve more precise, effective, and clinically translatable treatments for inflammatory and immune-mediated diseases.
